# There and back again: dynamics of temporary labor migration, insights from rural India

**DOI:** 10.3389/fsoc.2024.1422602

**Published:** 2024-08-06

**Authors:** Bernard Attah-Otu, Angan Sengupta, Tony McAleavy

**Affiliations:** ^1^Amrita School for Sustainable Futures, Amrita Vishwa Vidyapeetham, Amritapuri, Kerala, India; ^2^Amrita School of Business, Amrita Vishwa Vidyapeetham University, Bengaluru, Karnataka, India; ^3^Fire and Emergency Management Program, Division of Engineering Technology, Oklahoma State University, Stillwater, OK, United States

**Keywords:** temporary labor migration, internal migration, migration arrangement, destination conditions, precarity, tribal household, India

## Abstract

Temporary labor migration is a household phenomenon among rural communities in India. This study seeks to understand the subjective experiences influencing the temporariness of labor migration among internal migrants in India by examining various factors such as migration conditions, motivation, migration arrangements, coping and adaptation strategies, and determinants of stay. To achieve this objective, the current qualitative study utilized 14 in-depth interviews and 2 focus group discussions to investigate the temporary nature of labor migration among internal migrants in India. Our findings reveal that migration decisions are rational choices made collectively at the household level, considering socio-economic outcomes. We also find that social networks and contractors facilitate migration arrangements and job connections, and migrants employ various strategies to reduce costs and cope with expenses in urban areas. However, migration destinations often fail to meet migrants’ expectations, exposing them to low-wage employment and precarious working and living conditions, which are detrimental to their health. Limited housing and sanitation facilities further contribute to the challenges faced by migrants. Work conditions, including poor wages and high job demands, also affect their well-being. These findings highlight the need for improved support systems that address accommodation challenges, work conditions, and the overall welfare of labor migrants.

## 1 Introduction

Temporary labor migration (TLM) is a global phenomenon observed for centuries and occurs both domestically and internationally. Labor migration is driven by individual and household aspirations to enhance their capabilities and improve their socioeconomic well-being (De Haas, 2021). The expansion of TLM is a significant feature of the global economy in both developing and developed countries. Migration plays a crucial role in the service and agricultural sectors in many developing countries as it contributes to the workforce, sustains livelihoods (Khatri et al., 2023) and engenders human capital with over, 164 million migrant workers worldwide (ILO, 2021). Although TLM is viewed as a win-win situation for both the sending and receiving communities, it can, however, have an adverse economic effect on the sending communities (Bhagat and Keshri, 2020).

Temporary migration pathways have become fragmented, resulting in multiple non-linear movements, both internationally and internally. International labor migration takes place across countries, while the latter occurs within the country. Such migration is especially common among developing countries in Africa, South America, and Asia. Temporary labor migrants can, however, find themselves in low-skill and low-paying jobs that are short-lived but essential for addressing polarized labor market (Triandafyllidou, 2022). International migration is encouraged by developed countries, particularly for high-skill migrants, and often comes with an option for permanent resettlement. The nature of migration, whether regular or irregular, as well as the skills of the migrant, influence the type of employment at the destination place.

Temporary labor migration is crucial for livelihoods and economic development within South Asia, with significant movements observed among member states (Bryan, 2021). It contributes to income generation, employment opportunities, and economic returns for households (Deshingkar et al., 2006; Sathyapriya et al., 2022), while also fulfilling personal aspirations, and promoting capability development (De Haas, 2021). In India, temporary labor migration plays a prominent role in the economy, particularly with mass mobility from rural to urban areas. Recent trends have shown dynamic flows of temporary, seasonal, and short-term migration movements related to employment (Bhagat, 2010). Rural communities frequently engage in temporary labor migration to urban areas, seeking economic and material gains, notably within agricultural and farm-related enterprises located in urban areas. Migration conditions, however, often fall short of migrants’ expectations, with limited opportunities, low pay, and poor work and accommodation conditions at the chosen migration destinations (Kapilashrami and John, 2023).

The shift in mobility has prompted academic scholars to examine not only migration scenarios but also factors such as labor migration motivation, decisions, determinants and the impacts on both the destination and origin place (Srivastava, 2020). However, migration decisions are made at the household level and play a crucial role in deciding who migrates from the household, the associated commitment of the household, and offering households or individuals the opportunities. Understanding the linkage between migration decisions, migration processes, opportunities, and migration conditions offers insights into the root cause of the temporariness of labor migration.

Prior literature on temporary labor migration in India has predominantly focused on socioeconomic factors as the main determinants (Keshri and Bhagat, 2010, 2013; Dodd et al., 2016; Panda and Mishra, 2018; Sanyal and Maity, 2018; Sucharita, 2020; Rajan et al., 2023). Previous studies have analyzed temporary migration primarily as an economic phenomenon driven by financial incentives and the low socioeconomic status of rural areas, leading to its being viewed as a survival strategy for rural households in India (Keshri and Bhagat, 2013). Similarly, Dodd et al. (2016) supported this view, suggesting that temporary labor migration is propelled by historically disadvantaged caste groups seeking to cope with socioeconomic hardships within rural communities in India.

In their review, Panda and Mishra (2018) categorized factors affecting temporary labor migration in India into five main areas and sixty sub-factors, covering economic, social, environmental, psychological, and policy-related aspects. Sucharita (2020) investigated the socioeconomic determinants of temporary labor migration across 12 villages in Jharkhand, highlighting factors such as land ownership (or lack thereof), caste, and poverty as primary drivers among rural communities in the study area.

Despite the increasing body of literature on temporary migration in India, there remains a gap in research focusing on the socioeconomic determinants within households. Limited studies have examined the complexities of migration at both the household and destination levels that influence temporary rural–urban migration in India.

Amidst increasing evidence of temporariness and frequent return migration (Srivastava, 2020), there are gaps in understanding the root causes leading to the rise of temporary labor migration in India. This study aims to address this gap by critically evaluating migration dynamics at the place of origin and migration decisions at the household level and at the destination place among returned migrants in tribal communities in the Kattiwada Block of Alirajpur, Madhya Pradesh, India. Consequently, the study addresses the research question: How do household-level migration decisions in tribal communities of Madhya Pradesh, India, shape temporary labor migration patterns, and what are the key factors influencing migrants’ experiences, including their motivations, coping strategies, and return migration decisions?

The choice of Kattiwada Block in Alirajpur District, Madhya Pradesh, is driven by its status as the most poverty-stricken district in both the state and India, as highlighted in the Government of India Census of 2011. This region, primarily inhabited by Adivasi tribal communities, faces significant challenges, ranking lowest in the Multidimensional Poverty Index, with a high prevalence of Scheduled Caste and Scheduled Tribes (ST) (Tharu and Yadav, 2019). Tribal communities in India are characterized by high rates of occupational transition and mobility, stemming from their inherent socio-economic vulnerability (Shukla et al., 2016). Moreover, research into temporary labor migration scenarios in these areas has been lacking. Therefore, Kattiwada Block presents an ideal location for studying how the temporary nature of labor migration is influences across communities in this area.

This study addresses the noted gaps in the understanding of migration dynamics at the place of origin, migration decisions at the household level, and the influence of gender on those decisions. We also evaluate migration arrangements through social connections or contractors, migration conditions, coping strategies, adaptation, and the factors that determine longer stays or early returns to the place of origin. This study, therefore, contributes to the literature on temporary labor migration in several ways. Firstly, this study contributes to our understanding of the impact of rural household roles on the migration process spanning from migration decision to migration arrangement at the place of origin. Stark (1991) proposed that migration decisions are taken collectively as a household, considering the costs and benefits of the migration process as a safety net for the household.

Secondly, we analyze the migration arrangements from the place of origin, the coping and adaptation processes on transit, and the adaptation process at the destination place in three areas that cover job connection, travelling arrangements, and adaptation at the destination place. In this way, we offer a deeper insight into the different scenarios and decision processes that occur during transit among migrants. Thirdly, a key contribution of our study is in demonstrating the links between migration conditions at the destination place and family obligations at the place of origin. These are key determinants that influence the individual or household duration of stay which denotes temporariness in migration. We collected household data from household members and returned migrants on migration decision, on the role and contribution of the migrating household members to the left-behind families and how their desire to fulfil family duties and obligations influenced their duration of stay at the migration destination.

Traditionally, rural households are predominantly engaged in labor-intensive agriculture. However, the rational choice to send family members for employment implies a shortage in the agricultural sector (Harris and Todaro, 1970); thus, family members are obligated to return to their primary origin during certain phases, such as planting and harvesting, that are labor demanding. Finally, our study contributes to the literature by demonstrating how different migration conditions and experiences influence temporality among labor migrants in India by analyzing dwelling and work conditions, and rapport with employers as other crucial factors that influence migration duration.

This paper is structured into six different sections. The first section is the introduction, which discusses the background of the study, research gap, and prior literature on temporary labor migration. Following the introduction, is the theoretical background, where we the discus the new economics of labor migration theory as the theory guiding this study. In the third section, we present the methodological steps of this empirical study. Outlining the data collection methods, sampling technique, and data analysis. Thereafter, section four present the findings of this study on migration aspirations and household decisions, migration arrangements, migration conditions, and determinants of staying at migration destination. Section five discusses the findings and finally, section six concludes the study while section seven highlights the study limitations and recommendation.

## 2 Theoretical background

Migration has been extensively studied through various theoretical lenses, particularly at the meso-level, where migration is viewed both in terms of causes and perpetuation. Several theories have been adopted to examine migration, whether in the context of international migration flow or rural-to-urban migration flow (Ravenstein, 1889; Lewis, 1954; Lee, 1966; Todaro, 1969). This study builds upon the contributions of the new economics of labor migration (NELM) theory, which examines migration decisions at the household level.

The NELM theory, as proposed by Stark (1991), suggests that migration decisions are not made solely by individuals but are collective decisions made by households. The primary aim of this collective decision is to maximize income and minimize risks in response to economic failures, market failures, and vulnerabilities at the place of origin, with the ultimate goal of improving the household conditions. Unlike neoclassical theories that focus on individual income maximization, NELM emphasizes household-level choices in determining who migrates, under what conditions, and for how long. This approach aims to optimize the welfare and well-being of the entire household by diversifying income sources from household members working in different locations (Massey et al., 1993; Taylor, 1999; Hagen-Zanker, 2008; De Haas, 2010). While most previous migration theories (e.g., Lewis, 1954; Todaro, 1969; Harris and Todaro, 1970) ignored or remained silence on the role of remittance as income for the migrant household, the NELM views remittances as the most crucial and impactful aspect of migration to the migrant household left-behind. Remittances play a crucial role in coping with economic challenges and risk reduction for the household.

**Figure 1:**
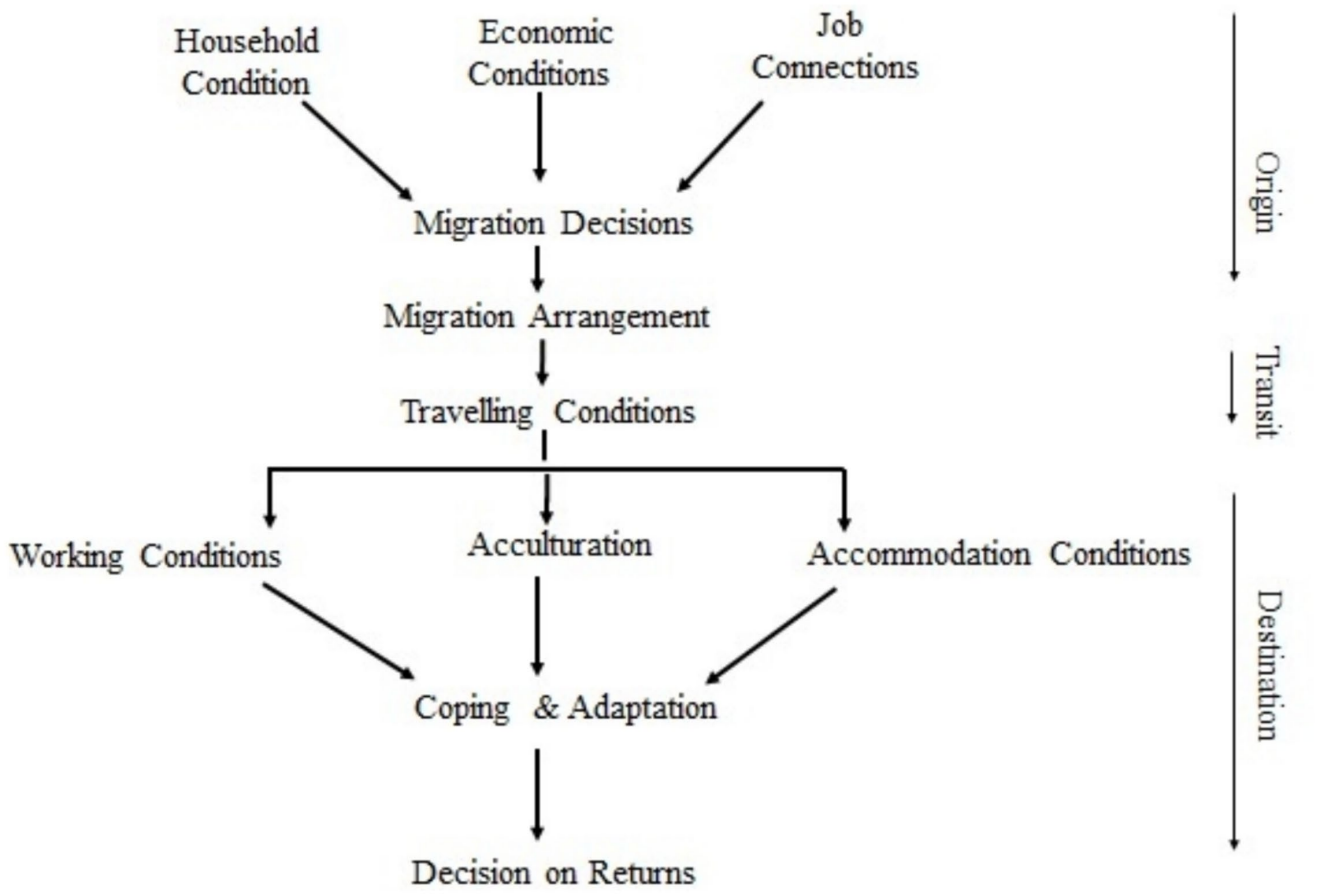
Flow chart of migration process. Source: authors own work.

Building upon the NELM theory, we developed a household migration process flow mechanism (Figure 1), which shows that the migration process is initiated from the place of origin to the destination, as proposed by Stark (1991). The initiation phase of migration begins at the household level, driven by economic conditions that motivate migration aspirations with the prospect of improvement. However, these aspirations might be hindered by factors such as distance, intervening obstacles, or personal circumstances (Lee, 1966). These challenges prompt households to make collective efforts, rather than individual decisions, to facilitate the migration process through social networking and family support from the origin to the destination (Figure 1). Migration journey is aided and supported by the household to facilitate smooth mobility forging to destination. Additionally, we present in our flow mechanism conditions at destination that influences and determine temporariness in labor migration

## 3 Methods

### 3.1 Research design

This study adopts a qualitative interpretivist phenomenological approach, comprising 14 semi-structured in-depth interviews focusing on migration life narratives and two focus group discussions involving 6–8 participants each (Bell et al., 2019). These discussions were conducted within four purposively sampled tribal communities between August and November 2022 (Bryman, 2012). Qualitative design was chosen as it aims to understand the lived experiences of individuals who have undergone a particular situation or phenomenon (Spencer et al., 2014; Neubauer et al., 2019). Phenomenology, as a research approach, typically explores the “why,” “what.” or “how” questions. This design was, therefore suitable for exploring the lived experiences of recently returned temporary labor migrants at their place of origin.

### 3.2 Database

A snowball sampling technique was employed due to the unfamiliarity of the study location and terrain to the researchers, and as per qualitative research norms the study sought theoretical saturation (Saunders et al., 2018). This technique, of snowballing, is widely used for such purposes as it serves as a referral system (Noy, 2008; Naderifar et al., 2017), A local contact was identified to help refer the researchers to appropriate members of their community based on their understanding of their community, local conditions and the needs of the research questions. To ensure representativeness, we requested detailed descriptions of each participant’s migration destination, the nature of work engaged in during migration, and frequency of migration. By selecting participants from four different communities and various migration destinations, we captured diverse individual and household experiences. This approach ensures triangulation and strengthens the study. However, such qualitative research has limited representativeness, meaning the posited findings are applicable to similar socio-cultural and economic context only (Maxwell, 1992), As such, we can only generalize this study findings to the Indian context. After each interview, participants were asked to refer the research team to other potential participants. This approach facilitated the selection of migrating household members, allowing the study to collect firsthand narratives, perceptions, and meanings attached to their migration experiences.

Ethical approval was obtained from the Institutional Ethical Committee, Institute of Medical Sciences Healthcare, Education and Research. Each participant was presented with an informed consent form, printed in English and translated into Hindi, at the beginning of each interview and focus group in accordance with the principles of ethical research.

### 3.3 Data collection

Data collection utilized in-depth interviews, field notes, and observation from August to November 222 in four tribal communities in the rural Kattiwada block, Alirajapur district, Madhya Pradesh, India. The lead author conducted a total of 14 in-person, in-depth interviews. All 14 participants were labor migrants who had recently returned to their village following a brief period of migration. Thirteen participants had returned from different migration destinations, primarily engaged in agricultural plantation work (such as banana harvest, transportation, coffee, or paddy cultivation) and construction work in the neighboring state of Gujarat. One participant migrated only within their district to maintain close family ties through monthly visits. Informed consent was obtained from all participants, with signed consent forms collected at the end of each interview.

Following saturation from the in-depth interviews, we conducted two focus group discussions (6–8 participants), one with men and another with women in two separate communities with non-migrant’s household individuals to gather information on household migration scenarios. Participants for the focus group discussion were purposefully chosen from different household in each case to obtain different household migration perspective from individuals who have not been migrating from their household (Krueger, 2014). Group discussions centered on household migration scenarios, migration discussions, choices or decisions, and the support offered to intending migrant household members. Selecting several individuals from different households for the group discussion ensures representativeness, captures diverse household perspectives, and validates the data obtained during the in-depth interviews (Duggleby, 2005). Additionally, conducting separate group discussion for the men and women ensures that every participant has the opportunity to contribute during discussion and is not silenced by cultural norms or intimidation.

### 3.4 Data analysis

The interviews were transcribed, forming a corpus of 16 transcripts (14 interviews and 2 focus groups), and the textual data were cleaned and analyzed. Coding of the textual data was conducted inductively through a line-by-line thematic iterative approach (Braun and Clarke, 2006), using the Dedoose qualitative data analysis software (Salmona et al., 2019; Huynh, 2021). Our study focuses on the migration process, including household migration decisions, work connections, and migration arrangement decisions at the migration destination that influence coping strategies determine the length of stay. Our coding focuses on narratives of experiences and meanings referencing a particular migration act, process, or experience both at the origin and destination. Clear patterns and similarities began to emerge during interview 11, meaning that theoretical saturation was achieved (Saunders et al., 2018). Similar codes were categorized and merged into clusters of themes that function as descriptive narratives of the participants to inform discussion (Beck, 2019). Table 1 depicts four themes. Three themes emerged from the household-level decisions at the place of origin covering sub-themes that include migration decision, job connection, and travelling arrangement. The fourth theme emerged as narratives and experiences at the migration destination, which includes various determinants related to duration of stay at migration destination, such as family duty and obligation, rapport with employer and work condition, and housing arrangements that cover coping and adaptation.

**Table 1:** Themes and sub-themes emerged from data analysis.

Themes	Area of focus of sub-themes
Migration dynamics	Household migration decisions
	Gender migration
Employment/Job connection	Social networks
	Contractors
Migration arrangement	Group travelling
	Support with food items from the village
Determinants of duration of stay at migration destination.	Family duty and obligation
	Rapport with employer and work conditions
	Availability of accommodation

For discussion purposes, the term ‘narrative’ was used to describe participants’ descriptions of migration events, representing their individual experiences and perceptions. Furthermore, Maxwell’s (1992) concept of descriptive, interpretive and theoretical validation were applied to ensure qualitative rigor within the research design and conduct of the research.

## 4 Results

In this section, we present the results of our findings from qualitative data analysis. Our thematic data analysis generated four different themes and sub-themes (Table 1). The first theme household migration decisions with two sub-themes “household migration” and “gender migration” explores households level migration decisions and how gender dynamics influences and shapes these decisions. The second theme “employment and job connections” focuses on job connections at place of origin, employment opportunities at destination and how social networking aid facilitates the migration process. The third theme addresses how migration arrangement is made at the household level and the choices taken before embarking on the migration journey. These themes also highlight collective efforts of the household to ensure a smooth migration process of the intending migrating household members. The final themes explore migrants’ conditions at their destination. We also address issues such as employment conditions, housing and migration experiences at their destination locations.

### 4.1 Household dynamics and migration decision making

Migration serves as a coping and adaptation strategy for rural households, and the decision of who migrates and who stays behind is a collective choice made by the household (Massey et al., 1993; Bahinipati et al., 2021; Sarwary et al., 2022). The size of the household plays a significant role in determining the migration decision, as larger households tend to find migration more beneficial and profitable. Consequently, rural households with larger sizes are more likely to send a higher proportion of their members for short-term labor migration.

During an in-depth interview, a participant explained how household size influences migration decisions:

The number of people that migrate depends on the number of people in a particular household; if there are more people in the household, then more people will migrate, and one married son will stay back in the village to care for the household, farm work, and livestock (In-depth interview narrative).

In cases where the household head is also migrating, they decide which children should accompany them. If the household size is small, they might choose one child, whereas, in larger households, multiple children may migrate.

A male household head shared his experience during an in-depth interview:

I made the migration decision. Here in my house, we are four; two people go out for migration while two others remain behind. I went to migration with my daughter while my wife and son stayed back in the village to care for the farm and animals. At first, I migrated alone, but after migrating for some time, I became familiar with the situation there (at the migration destination) and felt comfortable that it was safe to bring my child here. Later, I decided to bring my female child with me here to the migration place, where she works with me as a laborer. She supplies bricks and mixes concrete for me (In-depth interview narrative).

Migration scenarios vary for different household situations. In households with aged parents or grandparents, the eldest married children, the youngest unmarried male child, and adult unmarried females in their mid-twenties tend to migrate, while a married son stays back to assist with agricultural work, livestock care, and housing. These households observe permanent or longer-term migration, with the migrating children visiting home once or twice a year and maintaining connections through remittances, except in cases of severe illness or family deaths.

Another household head explained this situation during an in-depth interview:

The parent decides who should migrate if they should stay behind, or if they should send or allow their girl child to migrate. This is very important in the case of a female child. For male children, there is no such household decision. They usually express their decision and then move on with their peers or relatives. Family members that do not migrate take care of the house, livestock, and other family members (In-depth interview).

In nucleated households, mass migration is common, especially among young families who view migration as a promising enterprise. Limited access to land, water sources, and extended periods of off-season monsoon rain have further hindered rainfed agriculture in these areas, leading to the migration of husband, wife, and 2–3 children. Short-term migration is prevalent among this group, as they often return to their communities quickly.

A participant explained this situation during an in-depth interview:

My wife and I have two sons. We have a plot of land that is far away, and there is no bore-well around the farm plot. Crop yield here in the village is low, and we only get money from the farm every 3–4 months, so we just lock the house and migrate with the children. We do not keep livestock because there is no one to take care of them when we go away for migration (In-depth interview).

Gender also plays a crucial role in migration decisions (Lee, 1966). In patriarchal societies such as India, women have limited decision-making authority, resulting in restricted freedoms in certain aspects of life. Consequently, women residing in rural areas face greater challenges in migrating compared to men, male youths, or adolescents. Household migration choices are also influenced by gender. Participants disclosed that the migration of female children is more challenging for households than the migration of male children. Parents are reluctant to send their daughters alone without the security of a male companion such as a sibling or a husband. Parents fear for the security and safety of the daughters, as most parent fear believe they are not safe away from the family and protection by a male family member at during transit and at the migration destination. The level of restriction is relatively lower for married women as they can freely migrate with their husbands. However, unmarried girls encounter enforced migration decisions and are unable to migrate alone; they must always be accompanied by a male family member or a close extended relative.

During the migration process, both male and female children are required to seek permission from their parents before embarking on their journey. However, the decision-making power lies predominantly with the parents, particularly in the case of female children. Unlike male children who generally express their migration decisions and proceed with their peers or relatives, female children face different circumstances and require approval from their parents before migrating. In most cases some, fathers typically inquire about the conditions of the destination from individuals familiar with the location before granting permission for their children, especially daughters, to migrate. Furthermore, parents insist on the presence of a trusted relative or acquaintance from their village in the migration destination before allowing their child to proceed. Without such assurances, parents are reluctant to send their children to that particular place (as mentioned in an in-depth interview narrative).

### 4.2 Social networking among migrant communities

Social networks for job connections have been prevalent in the field of migration for several decades (Poros, 2011). Recent literature on migrants and migration networks (Bilecen et al., 2018; Lubbers et al., 2020) reflects a growing interest in studying migrant networks among academics and policymakers. Temporary labor migrants often come from low socioeconomic backgrounds and possess limited skills necessary for optimal performance. The evidence suggests that networking among migrants arises due to existing inequalities in accessing the labor market (Behtoui, 2008). raising questions about the location, formation, maintenance, and utilization of networks to secure specific job opportunities. Family and friend ties within the community are crucial for the rural household and serve as the rural–urban bridge for job connections. This ensures that information flow from their peers and contacts in urban areas is constant and consistent regarding jobs openings in the urban labor market. It also connects the community with support systems that ensure that intending migrants secure the right jobs, wages, and employment conditions, including housing, before they begin migration process. At the community level, information about job availability in the urban area is passed across households among kin and close friends who are perpetual migrants and aspiring youths. However, the migration of a household member depends on the nature of the job available, the migration destination, and the willingness of other family and community members to migrate to a particular destination.

Among households, our findings indicate that networking at the grassroots level (origin) occurs through two primary channels: networking with contractors and peer connections.

Contractors are community members who have migrated for an extended period and have established relationships and connections with farm owners, construction sites, or production facilities. To secure a job in a particular location, we contact people from our village who are already there and express our interest in finding work. If a job is available, they arrange it for us; otherwise, we reach out to other contacts in different places, and if there is work, they help us secure a position. Before going to the workplace, we receive information about the conditions, wages, and other relevant details. Nowadays, it has become easier to inquire about job availability in different places through phone calls, thanks to social media and mobile phones (In-depth interview narrative).

Another strategy of recruitment is through peers and kins at different migration destination.

Many individuals from our village work outside, some as supervisors who have leased agricultural fields. When they require labor or assistance, they call people from our village to join them, and once they arrive, they are employed to work in the fields (In-depth interview narrative).

The participants also emphasized the importance of working conditions and accommodation in the decision-making process related to job connections through networking. Considering factors such as wages and housing is integral to the prospective migrant’s perspective on the economic benefits associated with migration.

Digital media has made our work easier. When there is a demand for labor, contractors call and inform us about their requirements. They provide details about the pay and the nature of work and housing and we have the freedom to accept or reject the offers based on our preferences (In-depth interview narrative).

### 4.3 Group migration and travelling arrangements

Temporary labor migration is widely practiced among rural households that seek to improve their socioeconomic conditions by working in urban areas. However, most labor migrants engage in temporary migration because they are not willing to permanently settle in urban areas and give up their homes in their place of origin. The desire for economic gains rather than a permanent move drives the temporariness of migration in India, as migrants maintain roots in their place of origin. The term “Pardesh chalo” (Roy et al., 2015) is a popular song among labor migrants that signifies “let us go to a foreign land for livelihood” and can be interpreted as “let us go, make money, and come back home to enjoy.”

Considering the temporary nature of rural–urban mobility, migration arrangements for household members are made based on temporary modes. Intending migrants often prefer to return early after migrating due to family obligations or due to the work and accommodation situation at the destination. Migration decisions are typically made collectively by the household, and they often send two to three household members to different destinations or small destinations as a security measure and to diversify their sources of income.

One participant describes their migration arrangement:

“After making contact and securing the job, we wait and arrange with everybody who will be travelling to the same place. Then, we travel in groups with members from our house or with members from the same village. If there is no group of people leaving from the same village or area, parents do not allow their children (boys or girls) to leave the village for labor work” (In-depth interview narrative).

During migration process, food items and cooking utensils are taken from the village to the migration destination. Migrants carry rice, wheat, and other food items that they need to make meals at their destination. These food items serve them and their families for some time until they are able to earn their first wages to support their purchase of additional food or other necessary items. Taking food items from the village to the migration destination is a common practice among migrating households, as it helps mitigate expenses at the destination.

### 4.4 Determinants of stay at migration destination

Participants describe their desire to fulfil family obligations at their place of origin was a major reason for their short-lived migration. Family plays a crucial role in the migration decision, with arrangements often centered around remittances for home care, repayment of migration loans, and mutual support between migrating and left-behind family members. Migrants may need to return quickly to their place of origin to provide support or participate in family work or farming activities. Mothers, in particular, tend to maintain close ties with their children through remittances for home care and education.

One participant shared their experience:

When I migrate, the duration of my stay at the migration place depends on the work I have in the village. If there is work in the village, I return back quickly, and if there is no work, I stay longer. Whenever work arises in the village, I go there and finish it, then return quickly to the migration place (In-depth interview narrative).

For many men family obligation also extends to duties to spouses and children left behind as a trigger for early return to the place of migration. A male migrant may consider his wife and children’s welfare and security which also motivate his early return. Often, internal migrant labour remittance to his family is facilitated by a close friend or returning community member that takes the cash to the migrant family back home, however, in certain cases, the migrant inability to remit cash to his immediate family also acts as a push to return home early.

Sanja (a male migrant of 38–40 years) who migrate only within his district explain:

I migrate only within my district because I do have a wife and three young children. I have just come back after 22 days to see my family. When you have a family that you leave behind with young children, it is difficult to stay back. I will go back (migrate) after 3–5 days here in the village (In-depth interview narrative).

Maintaining a good rapport with employers and supervisors is crucial for the duration of stay at the migration destination. The quality of the employer-employee relationship can influence whether the migration is temporary or permanent. In India, labor migration tends to be more temporary due to poor work conditions and strained relationships between labor migrants and their employers.

One participant shared their experience:

When we work at a migration place, the duration of our stay depends on the place and the employer. If the employer treats us well, we will stay longer. Otherwise, we will leave quickly and return to the village. When there are too many restrictions, when the supervisor does not allow us to rest or keeps shouting at us, we do not feel happy and are forced to leave early. Some employers with plenty of money offer good working conditions, but they can be aggressive, always complaining about your work, and strict about working hours.

Working conditions also play a significant role in determining the duration of migration. Labor migrants often work in precarious and vulnerable conditions, deprived of proper rest and job security (World Bank, 2016).

One female participant shared her experience:

I work in construction with my husband. Sometimes, the supervisor gives us work and expects us to finish it within a short time. The supervisor constantly checks on us, and if we fail to complete the work, he shouts, quarrels, and calls us lazy. It becomes difficult to concentrate, and I get angry. Sometimes, I shout back and try not to cry. If it were not for the need to earn money, I would not go back there again (In-depth interview narrative).

## 5 Discussion

Ethnographer James P. Spradley (1933–1982) famously stated, “I want to understand the world from your point of view. I want to know what you know in the way you know it. I want to understand the meaning of your experience, to walk in your shoes, to feel things as you feel them, and to explain things as you explain them. Will you become my teacher and help me understand?” This study sought to make sense of participants subjective lived experience (Spencer et al., 2014; Neubauer et al., 2019). The first two categories occur at the place of origin, while the latter two pertains to the place of destination. Each set of factors (Figure 1) was carefully categorized based on the observed meaning and important converged through body language and emphasis during participant interviews (Kvale, 1996; Qu and Dumay, 2011).

This study focused on men and women who primarily migrate for labor work in construction and agricultural plantations, as well as non-migrating household members who play a role in the migration process (Stark, 1991; Massey et al., 1993). The findings revealed that household decisions regarding migration are rational choices made collectively, considering the socio-economic outcomes associated with migration. These findings align with Stark (1991) new economics of labor migration theory, which emphasizes that migration decisions are made at the household level.

Furthermore, the results indicate that family migration arrangements and job connections are predominantly facilitated through social networks and contractors, which aligned with the social network theory (Munshi, 2014). Additionally, migrants employ various strategies, such as makeshift accommodation, savings, and communal cooking, to reduce migration costs and cope with the high expenses in urban areas. Finally, this study identified several key factors that influence the temporary nature of labor migration, including family obligations, housing availability, rapport with employers, and working conditions at the destination. This study is, therefore, unique in its comprehensive exploration of the migration cycle from the point of origin to the destination. Nonetheless, the posited findings align with experiences reported in other labor migration scenarios (Mak et al., 2021).

Migration serves as livelihood strategies and coping mechanisms for households in the study area. Hence each household typically has at least 2–3 family members migrating at different times throughout the year. Within the household, migration choices and decisions are multifaceted and made for the overall benefit of the entire family (Adams, 2004; Mulder, 2007). Our finding also shows that preparations for migration are made well in advance of the migrant’s departure (Jasinskaja-Lahti and Yijal, 2011; Tabor and Milfont, 2011), and family members take on the responsibility of financing the migration process through various means such as pooling resources, seeking donations, or borrowing from neighbors According to Stark’s (1991) new economics of labor migration theory, decisions on migration are rational choices made by households that consider both costs and benefits. Consequently, households base their migration decisions on what they perceive as the most advantageous alternative to address low agricultural output (Ellis, 1998). They select migration destinations and work opportunities based on the potential outcomes, including remittances, savings, and skill acquisition after covering expenses at the destination.

Among rural households, migration choices and decisions are also influenced by who within the family is migrating, and consider factors such as family migration and individual migration, as well as gender and age. In terms of gender-related migration, male children aged 15–18 receive greater preference from the household as most parents do not deem it fit to allow their female children to be away from home where they are not monitored or safety provided for them by male household member. This can also be attributed to the patriarchal family system that allows the males more freedom while females are restricted to certain roles in the household. However, males do not only migrate more frequently but also tend to stay for extended periods at the destination, with fewer visits home except during peak agricultural activities. On the other hand, female children in the study area have limited opportunities for migration, except when accompanied by an elder sibling, parents, or a trusted extended family member. The freedom of females to migrate is typically achieved only through marriage, which allow them to accompany their husbands during migration, and also work under their guidance as support workers. These dynamics illustrate how intergenerational roles and the need for support systems for the elderly influence migration decisions. Furthermore, restriction on female migration not only constrains women’s aspirations and potential but also hampers their ability to develop skills, make independent choices, and participate more fully within society (De Haas, 2021). This demonstrates the gendered nature of migration decisions and the cultural norms shaping them.

Harris and Todaro's (1970) two- sector theory of migration posits that wage disparities between rural and urban regions serve as the primary driver for rural-to-urban migration. The availability of job opportunities is predominantly concentrated in urban areas, which necessitates the relocation of residents from their rural dwellings to urban centers in search of employment (Harris and Todaro, 1970). Migration costs and benefits tend to rise with distance, prompting rural individuals to rely on social networks and peers for job connections. Intervening factors, such as distance (Lee, 1966), play a crucial role in the migration process, particularly for first-time migrants, women, and children, who often depend on relatives and extended family members to guide them to their destination. Given the limited social and human resources in rural areas, household members support one another through forming group and utilizing social networks, thereby reducing the risks and vulnerabilities associated with the migration journey.

We noted that migration destinations often fail to meet migrants’ expectations, exposing them to low-wage employment and precarious working and living conditions that have significantly detrimental effects on their health. These findings align with previous studies, such as those by Khurana (2017) and Srivastava and Jha (2016), which observed precarity among labor migrants in India. According to Dako-Gyeke et al. (2023), labor migrants frequently employ various coping and adaptation strategies which were observed among unaccompanied migrant workers from West African countries notably Ghana. These strategies include seeking assistance from social networks, relying on migrant aid groups, utilizing available resources, and employing distractions and avoidance techniques. However, for internal labor migrants in India, their precarity stems from a lack of social skills and networks which restricts their social interactions with their immediate family and individuals they are familiar with at their destination. The absence of adequate accommodation and resting places creates significant stress for laborers engaged in strenuous work, as they are often expose to direct sunlight during 10-12 hours word a day, and are in small, makeshift sheds (Dhal, 2020).

Additionally, we found that low and irregular wages often prevent individuals and households from effectively utilizing their wages to fulfil their migration aspirations and enhance their capacity development (Srivastava and Jha, 2016). To make ends meet, migrants often share resources among kinsmen, family and friends, and cook in groups. While these practices enable migrants to save to some extent, they have adverse health effects due to reduced food consumption and inadequate nutrition, particularly when communal cooking arrangements replace individual or single-family meal preparations.

Furthermore, the well-being and economic welfare of children are also compromised, as there is limited food and nutrition available to meet their basic needs. Parents are absent during the day, so meals are prepared, relying on group provisions, occasionally supplemented with snacks for lunch. Children tend to consume their food before noon without adult supervision, leaving them hungry for a significant portion of the day until an adult family member returns to prepare another meal late at night, which can result in a gap of more than 10 h. This has had significant impact on children’s health, leading to malnutrition among migrant children (Ravindranath et al., 2019).

Temporary and short-span migration, along with early return, is common among internal labor migrants in India (Keshri and Bhagat, 2012, 2013). The desire to maintain family connections and fulfil obligations drives their willingness to return home. Migration decisions are made collectively within households, and migrating members provide support to those left behind through remittances or household farm work. Work support emerged as a major factor determining the temporary nature of migration, with migrants often returning during crucial agricultural operations such as land preparation, cultivation, and harvesting. This study also reveals that employers in the construction and agricultural sectors do not provide adequate housing and sanitation facilities for migrants. Limited access to adequate housing and sanitation was observed during their stay at migration destinations. The reason behind this limitation was not fully explored, but it is likely due to middlemen, contractors, and subcontracting processes prevalent in these sectors in India (Srivastava, 2020). For instance, laborers in the agricultural sector often hire additional laborers through a middleman or contractor. However, the middleman, who often has limited accommodation themselves, struggles to provide housing for the additional laborers. This situation arises when a laborer rent a plantation field but requires extra help, leading to a shortage of suitable accommodation for the requested laborers.

Work conditions play a significant role in the migrant’s experience. Previous studies such as ILO (2012) and Mak et al. (2021) have identified various stressors, including environmental stress (poor housing, overcrowding), workplace stress (poor conditions, low wages), family stress (children’s needs, safety concerns), and acculturation. We found that poor relationships with employers or supervisors also contribute to stress and the desire to seek alternative opportunities. Migrants reported facing intense pressure to complete tasks within short timeframes, which is consistent with findings among labor migrants in several other countries (Lee et al., 2012). Additionally, migrant laborers often engage in high-risk and injury-prone jobs without adequate health and safety training (Kaur, 2010).

## 6 Conclusion

This study employed a phenomenological approach to gain insight into the transient nature of labor migration among internal migrants in rural India by examining critical factors such as migration decisions, arrangements, coping strategies, and determinants of migration’s temporary nature. The study focused on both men and women migrating for work in construction and agricultural plantations, as well as non-migrating household members involved in the migration process.

Migration decisions were found to be rational choices made collectively at the household level, considering socioeconomic outcomes. Factors such as family obligations, housing availability, rapport with employers, and working conditions at the destination influenced these decisions. The study also highlighted gender disparities in migration decisions, with male children being favored for longer stays at the destination, reflecting cultural norms and intergenerational roles.

Social networks and contractors played significant roles in facilitating migration arrangements and job connections for labor migrants. To cope with high expenses in urban areas, migrants employed various strategies such as makeshift accommodation, savings, and communal cooking. However, migrants often faced precarious working and living conditions at the destination, affecting their health and well-being, leading to malnutrition among migrant children.

## 7 Limitations

All research has limitations; this study was no exception (Bell et al., 2019). Language barrier was a significant challenge as the principal investigator did not speak Hindi, the primary language of participants. Relying on an interpreter during interviews and discussions may also have introduced potential bias. However, to mitigate this, printed interview questions were provided to the interpreter, and responses were translated into English during the interviews to provide better insight and understanding and to aid notetaking. Additionally, the small sample size posed a challenge. However, whilst these are certainly limitations, the research design and conduct, pursuit of theoretical saturation and application of Maxwell’s (1992) descriptive, interpretive, and theoretical validity are in line with the norms and values associated with qualitative research (Yin, 2010).

### 7.1 Recommendations

The study emphasizes the need to address accommodation, work conditions, and overall welfare of labor migrants. Improved support systems are necessary to ensure their safety, health, and economic welfare. Providing adequate housing, sanitation facilities, and health and safety training are crucial steps. Interventions should also aim to address gender disparities in migration decisions, promoting equal opportunities for both male and female migrants to enhance equality, women’s empowerment, and economic development.

Further research is recommended to explore factors influencing migration decisions and the temporary nature of labor migration in different regions of India. Comparative studies across countries and contexts would contribute to a comprehensive understanding of labor migration dynamics. Investigating long-term effects of migration on migrants and their families, as well as socio-economic impacts on origin and destination communities, would inform policy development and interventions. Additionally, given the intensity of labor migration in the study area, future research should explore intergenerational aspect of labor migration with focus on sociohistorical context and why labor migration has persist in this area.

## Data availability statement

Due to the sensitive nature of the topic and the anonymity of participants, data for this study are not for sharing. Further inquiries can be directed to the corresponding author.

## Ethics statement

The studies involving humans were approved by Amrita Vishwa Vidyapeetham Institute of Medical Science, Healthcare, Education & Research, Institutional Ethics Committee. The studies were conducted in accordance with the local legislation and institutional requirements. Written informed consent for participation in this study was provided by the participants’ legal guardians/next of kin.

## Author contributions

BA-O: Conceptualization, Formal analysis, Writing – original draft, Writing – review & editing, Data curation, Investigation, Methodology. AS: Supervision, Writing – review & editing, Validation. TM: Writing – review & editing.
